# Octreotide for prevention of delayed bleeding after endoscopic mucosal resection for large superficial non-ampullary duodenal tumors

**DOI:** 10.1055/a-2798-2041

**Published:** 2026-03-19

**Authors:** Cecile Maxime Van De Leur, Leon MG Moons, Frank Vleggaar, Paul Didden

**Affiliations:** 1 8124 Gastroenterology and Hepatology, University Medical Centre Utrecht, Utrecht, Netherlands

**Keywords:** Endoscopy Upper GI Tract, Endoscopic resection (ESD, EMRc, ...), Endoscopy Lower GI Tract, Polyps / adenomas / ..., Quality and logistical aspects, Performance and complications

## Abstract

**Background and study aims:**

Effective strategies to prevent delayed bleeding (DB) following endoscopic mucosal resection (EMR) for large superficial non-ampullary duodenal tumors (SNADTs) remain limited. Octreotide exerts pharmacological effects that may influence hemostasis and thereby reduce bleeding risk. This exploratory study aimed to evaluate the association between postprocedural octreotide use and DB after duodenal EMR for large SNADTs.

**Patients and methods:**

This single-center, exploratory, retrospective study included all hot EMRs for large SNADTs ≥ 10 mm (2015–2024). Routine post-procedural intravenous octreotide (50 μg/h) was introduced in 2022, administered immediately post-EMR at 50 μg/hour during overnight observation. DB was defined as clinically significant bleeding within 30 days requiring prolonged hospitalization, readmission, or intervention.

**Results:**

A total of 107 SNADTs (median diameter 25 mm; interquartile range 15–40) were resected, including 33 treated with octreotide. Overall, DB occurred in 20% of patients (21/107), including 24% (18/74) in the control group and 9% (3/33) in the octreotide group. In logistic regression adjusting for lesion size and propensity score, octreotide showed a non-significant trend toward reduced DB overall (odds ratio [OR] 0.34; 95% confidence interval [CI] 0.08–1.17; *P* = 0.11). In the subgroup of lesions ≥ 30 mm, octreotide use was associated with a significantly lower odds of DB (OR 0.21; 95% CI 0.039–0.89; *P* = 0.045), whereas this association was not observed in smaller lesions.

**Conclusions:**

In this exploratory study, postprocedural intravenous octreotide appeared to be associated with a lower occurrence of DB after EMR for large SNADTs (≥ 30 mm). Larger, prospective studies are warranted to confirm these findings.

## Introduction

Superficial non-ampullary duodenal tumors (SNADTs) are rare, with a prevalence of 0.03% to 0.4%, and are often incidentally diagnosed during endoscopy. Endoscopic mucosal resection (EMR) has emerged as preferred treatment for removal of nonmalignant large SNADTs (≥ 10 mm) 1 . Due to the abundant vascular supply, thin wall, and potential toxic effects of bile and pancreatic secretions, EMR in the duodenum carries significant intraprocedural and post-procedural risks, particularly delayed bleeding (DB) 2 3 The adverse event (AE) rate further increases for giant lesions (≥ 30 mm), with a DB rate of up to 40% 4 5 .

Despite increasing interest in prevention of DB after EMR, evidence of effective strategies for SNADTs remains limited. Prophylactic clip closure of the post-EMR defect using clips or suturing devices, application of topical hemostatic agents, and use of cold EMR have been proposed as potential solutions; however, each approach has specific disadvantages or lacks proven efficacy 6 7 8 9 .

Octreotide, a synthetic somatostatin analog, reduces portal pressure through splanchnic vasoconstriction and inhibits exocrine pancreatic and bile secretions 10 . Although supporting evidence is still lacking, these effects may be beneficial in reducing risk of DB after duodenal endoscopic resection. Based on this rationale, intravenous (IV) octreotide has been routinely administered following duodenal EMR for large SNADTs at our center since 2022. This exploratory study aimed to assess whether postprocedural octreotide use was associated with reduced incidence of DB following EMR of SNADTs.

## Patients and methods

### Patient selection

This exploratory retrospective study was conducted on patients referred to the University Medical Center Utrecht for endoscopic resection of SNADTs between 2015 and 2024. Cases were identified through an electronic endoscopy reporting system search. Inclusion criteria comprised all standard hot EMR procedures performed for large SNADTs (≥ 10 mm). Patients diagnosed with familial adenomatous polyposis were also included. Involvement of the ampulla was an exclusion criterion. This study was approved by the Ethics Committee of the University Medical Center Utrecht (reference number: 24U/1354).

### EMR procedure

**Fig. 1:**
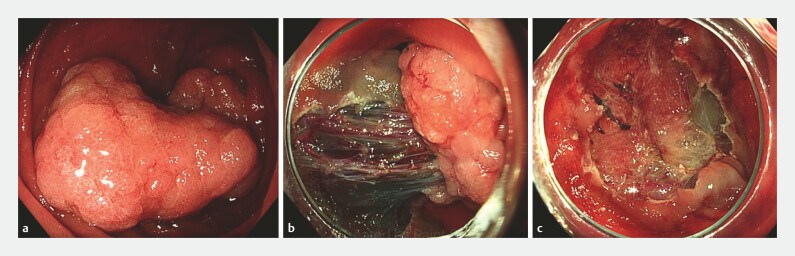
Piecemeal endoscopic mucosal resection (EMR) of a large superficial non-ampullary duodenal tumor (SNADT). **a** 60 × 40 mm sessile duodenal adenoma located at the transition of descending (D2) to horizontal part (D3), without clear optical features of malignancy. **b** Performance of hot EMR after submucosal injection with a solution containing gelofusine, indigo carmine, and epinephrine (1:100.000). Large vessels are seen in the submucosa. **c** Post-EMR defect showing complete removal of adenomatous tissue. Margin thermal ablation using snare tip soft coagulation was applied.

All endoscopic procedures were performed by three experienced interventional endoscopists (PD, LM and FV). Only standard lift-and-cut hot EMR was used, without applying the underwater or cold EMR technique. First, a lifting solution containing gelofusine, indigo carmine, and diluted epinephrine (1:100.000) was applied for submucosal injection. Subsequently, en bloc or piecemeal hot snare resection was performed using a 10- or 20-mm stiff snare (Captivator; Boston Scientific) ( Fig. 1 ). Intraprocedural bleeding was managed with snare tip soft coagulation (STSC) or, if unsuccessful, with hemostatic forceps. Adjunctive and adjuvant techniques such as argon plasma coagulation (APC) and STSC were applied at endoscopist discretion. Prophylactic clip closure at the post-EMR site was performed based on operator judgment. No predefined criteria were used, and closure was generally not attempted for large lesions (> 30 mm) due to technical limitations in achieving complete closure. Closure was considered complete when the distance between the clips was ≤ 1 cm without leaving large mucosal gaps (assessed by intraprocedural evaluation and if not reported by still image documentation).

Existing Dutch guidelines on antithrombotic therapy around endoscopic procedures were followed 11 . Only aspirin was continued. In cases involving other antiplatelet agents or dual therapy, the non-aspirin agent was discontinued 5 to 7 days prior to the procedure. Direct oral anticoagulants (DOACs) were withheld for 2 days and other anticoagulants for 3 to 5 days, with bridging therapy as needed. Antithrombotic agents were resumed 1 day post-procedure if no DB occurred.

Postprocedural care included a fluid diet overnight and proton pump inhibitor therapy with pantoprazole 40 mg twice daily during the first 24 hours, then continued at 40 mg once daily thereafter. No other acid-suppressive medication was prescribed. Most patients underwent one night of in-hospital observation. Octreotide was administered according to a revised protocol starting in March 2022 for all patients undergoing EMR, unless performed as an outpatient procedure. There were no changes to procedure technique. It was infused IV at 50 μg/h immediately post-EMR and continued for approximately 24 hours during the observation period. In cases of prolonged admission due to AEs, octreotide administration was extended at treating physician discretion. Post-treatment surveillance included endoscopy at 6 months to assess post-EMR scars for recurrent adenoma.

### Study outcomes

The primary outcome was occurrence of DB following EMR of SNADTs. DB was defined as bleeding occurring after the procedure, up to 30 days post-EMR, that resulted in an unplanned medical presentation such as Emergency Department visit, (prolonged) hospitalization (including need for transfusion), or reintervention (repeat endoscopy, angiography, or surgery) 12 .

Secondary outcomes included other AEs, such as perforation, re-endoscopy and endoscopic intervention after DB, and recurrence at 6-month follow-up.

### Statistical analysis

Because this study was exploratory in nature, no formal sample size calculation was performed. Continuous data are summarized as mean ± standard deviation or median with interquartile range (IQR), whereas categorical data are presented as frequencies. Statistical comparisons of continuous variables were performed using the Mann-Whitney U test. Categorical variables were analyzed using Fisher’s exact test or Pearson’s chi-square test, as appropriate. Statistical significance was set at *P* < 0.05. Statistical analyses were performed using IBM SPSS Statistics for Windows, version 23.0, and RStudio. To optimally control for confounding and minimize risk of overfitting in this retrospective cohort, a propensity score (PS) was constructed based on potential confounders associated with both octreotide use and DB: anticoagulant use, prophylactic closure, and lesion size. In the final logistic regression model, octreotide was the primary predictor, and both PS and lesion size were included as covariates. To explore potential effect modification by lesion size, an interaction term between octreotide and lesion size (< 30 mm vs. ≥ 30 mm) was included. Based on this exploratory interaction, subgroup analyses were performed for lesions < 30 mm and ≥ 30 mm.

## Results

### Patient, lesion, and procedure characteristics

Baseline characteristics of 89 patients who underwent EMR for 107 SNADTs are shown in Table 1 , with 33 procedures in the octreotide group and 74 in the control group. Two patients treated after the protocol change did not receive octreotide due to EMR being performed as an outpatient procedure. No significant differences were found between groups regarding American Society of Anesthesiologists score or antithrombotic therapy. However, median lesion diameter was significantly larger in the octreotide group (30 mm [IQR 20–50] vs. 25 mm [IQR 15–30]; *P* = 0.044). In 98% of cases, macroscopic complete resection was achieved, mostly through piecemeal EMR. Adjuvant therapy with STSC or APC was more common in the octreotide group (52% vs. 22%, *P* = 0.008). Complete prophylactic clip closure was similar between groups (46% vs. 39%, *P* = 0.184). Octreotide was administered for a median of 1 day (range 1–8).

**Table:** **Table 1** Baseline characteristics.

	Total	Control	Octreotide	*P* value
Patient characteristics*	**n = 89**	**n = 61**	**n = 28**	
Age, mean (SD), years	67.5 ± 9.2	67.6 ± 9.7	67.1 ± 8.6	0.586
Sex, female, n (%)	43 (48)	31 (50)	12 (43)	0.401
FAP or other polyposis, n (%)	8 (9)	6 (10)	2 (7)	0.289
ASA grade > 2, n (%)	23 (26)	19 (31)	4 (14)	0.138
Liver cirrhosis	2 (2)	1 (2)	1 (4)	0.517
APT/ACT, n (%)	23 (26)/18 (20)	18 (29)/14 (23)	5 (18)/4 (14)	0.282
**Lesion characteristics**	**n = 107**	**n = 74**	**n = 33**	
Location, n (%)				
D1+D1–2/D2/D2/3 + D3	12 (11)/58 (54)/37 (35)	8 (11)/43 (58)/23 (31)	4 (12)/15 (46)/14 (42)	0.459
Diameter, median [IQR], mm	25 [15–40]	25 [15–30]	30 [20–50]	**0.044**
Lesion size by group, n (%)				0.102
10–29 mm	58 (54)	44 (59)	14 (42)	
≥ 30 mm	49 (46)	30 (41)	19 (58)	
Paris classification, n (%)				
0-IIa/0-IIa + 0-Is/Is or Isp	49 (46)/14 (13)/44 (41)	32 (43)/8 (11)/34 (46)	17 (52)/6 (18)/10 (30)	0.138
Final histology, n (%)				
LGD/HGD/CA/unknown	79/26/1/1	58/14/1/1	21/12/0/0	0.219
**Procedure characteristics**				
Piecemeal/en bloc, n (%)	84 (79)/23 (21)	55 (74)/19 (26)	29 (88)/4 (12)	0.134
Macroscopic complete resection, n (%)	105 (98)	74 (100)	31 (94)	0.093
Complete prophylactic closure	44 (41)	29 (39)	15 (46)	0.184
10–29 mm	34/58 (59)	26/44 (59)	8/14 (57)	
≥ 30 mm	10/49 (20)	3/30 (10)	7/19 (37)	
Adjuvant STSC/APC, n (%)	33 (31)/10 (9)	16 (22)/8 (11)	17 (52)/2 (6)	**0.008**
Intraprocedural bleeding, n (%) ^†^	49 (46)	30 (41)	19 (58)	0.141
Octreotide duration, days (median) [range]	N.A.	N.A.	1 [1–8]	
Patient and lesion characteristics, procedural details, and prophylactic measures in the overall cohort and by treatment group. Values are presented as n (%) unless stated otherwise. Percentages for complete prophylactic closure are based on the total number of lesions in each size category (< 30 mm vs ≥ 30 mm). *At first procedure. ^†^ Intraprocedural bleeding was defined as persistent oozing or spurting of blood during the procedure that did not spontaneously cease within 60 seconds and necessitated endoscopic intervention. ACT, anticoagulation therapy; APC, argon plasma coagulation; APT, antiplatelet therapy; ASA, American Society of Anesthesiologists; CA, carcinoma; D1, D2, D3, first, second, and third of the duodenum; FAP, familial adenomatous polyposis; HGD, high-grade dysplasia; LGD, low-grade dysplasia; SD, standard deviation; STSC, snare tip soft coagulation.				

### Adverse events

AEs occurred in 22% of the 107 duodenal EMRs. DB was the most common AE, occurring in 21 of 107 cases (20%) ( Table 2 ). The number of DB events was lower in the octreotide group, with three cases among 33 procedures, compared with 18 cases among 74 procedures in the control group, although this difference did not reach statistical significance ( *P* = 0.054). No instances of DB were observed in patients who underwent simultaneous resection of multiple polyps. DB typically occurred within a median of 2 days post-procedure, with no difference in time to onset between the groups. Two of the three DB cases in the octreotide group occurred after discontinuation of octreotide administration. Repeated endoscopy was performed in 20 of 21 DB cases, and 15 required hemostatic intervention. Blood transfusions were administered in 16 of 21 DB cases. In the control group, 15 of 18 patients with DB received transfusions, whereas in the octreotide group, one of three patients with DB received a transfusion.

**Table:** **Table 2** Adverse events.

	Total (n = 107)	EMR (n = 74)	EMR + octreotide (n = 33)	*P* value
**Delayed bleeding, n (%)**	21 (20)	18 (24)	3 (9)	0.054
Onset, days [IQR]	2 [1–6]	2 [1–6]	2 [1,5–5,5]	0.753
Repeated endoscopy, n (%)	20 (95)	17 (94)	3 (100)	0.867
Endoscopic hemostatic intervention, n (%)	15 (71)	13 (72)	2 (67)	0.844
Embolization, n (%)	1 (5)	1 (6)	0	0.676
Transfusion, n (%)	16 (76)	15 (83)	1 (33)	0.128
**Perforation, n (%)**	3 (3)	2 (3)	1 (3)	0.332
Surgical intervention, n (%)	1 (1)	0	1 (3)	0.223
**Mortality, n (%)**	1 (1)	0	1 (3)	0.333
**Duodenal stenosis, n (%)**	6 (6)	4 (5)	2 (6)	1.000
Adverse events after duodenal EMR with or without octreotide. Values are n (%) unless stated otherwise; onset of delayed bleeding is shown as median [IQR]. *P* values from χ²/Fisher’s exact test or Mann-Whitney U test. EMR, endoscopic mucosal resection; IQR, interquartile range.				

A total of three perforations occurred among the 107 cases: two in the control group (2/74) and one in the octreotide group (1/33). Two of these perforations were intraprocedural: one was successfully managed endoscopically, whereas the other required surgical intervention, resulting in Intensive Care Unit admission and subsequent mortality. The remaining patient developed a delayed perforation 3 days post-procedure, which was treated conservatively with antibiotics. No AEs related to octreotide therapy were observed.

Logistic regression was performed to assess the association between octreotide and DB, adjusting for lesion size and PS ( Table 3 ). Octreotide showed a non-significant trend toward reduced odds of DB in the overall cohort (OR 0.34; 95% confidence interval [CI] 0.08–1.17; *P* = 0.11), whereas polyp size was independently associated with higher odds of DB (OR 1.10 per mm; 95% CI 1.05–1.17; *P* < 0.001).

**Table:** **Table 3** Logistic regression of delayed bleeding in overall cohort and lesion size subgroups.

	Factor	OR (95% CI)	*P* value
**Overall cohort**	**Octreotide**	0.34 (0.08–1.17)	0.11
	**Polyp size (mm)**	1.10 (1.05–1.17)	**< 0.001**
**Lesions ≥ 30 mm**	**Octreotide**	0.21 (0.04–0.89)	**0.045**
Logistic regression models of octreotide use and delayed bleeding after duodenal EMR in the overall cohort and subgroup by lesion size (≥ 30 mm). ORs with 95% CIs are shown. Models were adjusted for confounding using a propensity score based on antithrombotic therapy, lesion size, and prophylactic closure. CI, confidence interval; OR, odds ratio.			

**Fig. 2:**
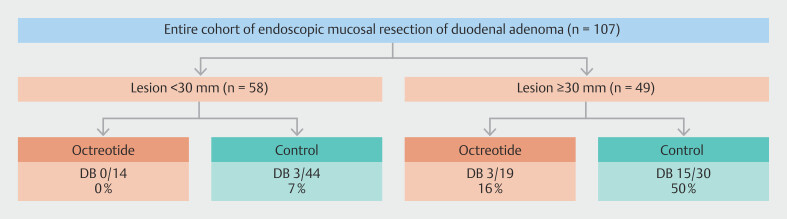
Rates of delayed bleeding across different subgroups. Flowchart showing the occurrence of delayed bleeding after duodenal EMR in octreotide and control groups, stratified by lesion size (< 30 mm vs. ≥ 30 mm). Sample sizes (n) and DB rates (%) are shown for each subgroup. DB, delayed bleeding.

Octreotide and lesion size (< 30 mm vs ≥ 30 mm) showed a non-significant interaction (OR 0.21; 95% CI 0.015–5.84; *P* = 0.28), indicating a potential difference by lesion size. Consistent with this observation, in an exploratory subgroup analysis of lesions ≥ 30 mm, octreotide use was associated with lower odds of DB (OR 0.21; 95% CI 0.04–0.89; *P* = 0.045) ( Table 3 ). In lesions < 30 mm, DB occurred in three of 44 controls (7%) and in none of the 15 octreotide-treated patients; logistic regression was not performed due to absence of events in the octreotide group. Fig. 2 illustrates the occurrence of DB stratified by lesion size.

### Long-term follow-up

A total of 80 patients underwent 6-month follow-up endoscopy at our hospital, showing local recurrence in 20 of 80 cases (25%). Nineteen (95%) of these recurrences were managed with repeat endoscopic resection, whereas one patient required surgical intervention due to endoscopic treatment failure. The remaining participants either had follow-up at their referring hospitals, declined surveillance, or have pending follow-up.

## Discussion

Duodenal EMR for large SNADT carries a high risk of AEs, particularly DB, highlighting the need for effective prophylaxis. In this exploratory study, octreotide use was significantly associated with a lower occurrence of DB among patients with large lesions (≥ 30 mm), indicating a possible role for octreotide as targeted prophylaxis in these high-risk endoscopic resections.

The potential beneficial effect of octreotide after duodenal EMR likely stems from its multiple mechanisms of action. First, octreotide reduces duodenal and splanchnic blood flow by inhibiting vasodilatory hormones, leading to decreased mesenteric perfusion, enhanced platelet aggregation, and stabilized clot formation 20 21 . This hemostatic effect may be further enhanced by suppression of gastrin, pepsin, and gastric acid secretion. Due to these physiological effects, octreotide has shown efficacy in both portal hypertension-related variceal hemorrhage and bleeding from small intestinal angiodysplasia 4 13 14 . Second, it inhibits bile secretion and pancreatic exocrine output, potentially reducing their harmful effects on the post-EMR duodenal defect 10 . A previous study reported more frequent AEs after EMR of polyps distal to the ampulla, suggesting that exposure to bile and pancreatic secretions may contribute to DB 15 .

Our study suggests that patients undergoing EMR for giant lesions (≥ 30 mm) may benefit from octreotide therapy. Lesion size is a well-established risk factor for DB, with higher bleeding rates reported in giant lesions 4 5 . In the subgroup of patients with lesions ≥ 30 mm, octreotide use was associated with lower odds of DB, suggesting a potentially protective effect in this high-risk population. This trend is encouraging, particularly given the limited prophylactic options currently available for large duodenal lesions. However, the wide CI, reflecting the small sample size and limited number of events, indicates that the precise magnitude of the effect is uncertain; therefore, these findings should be interpreted as exploratory. Several studies have suggested that complete closure of the post-EMR defect reduces DB risk 7 8 16 . However, achieving complete closure in the C-shaped, fixed duodenal anatomy, especially for larger defects, is technically challenging or even not feasible with through-the-scope (TTS) clips. Although suture-based techniques may offer a solution, their availability is limited and they significantly increase procedure costs 17 . Topical hemostatic agents have also been explored as a prophylactic option; however, a recent randomized trial failed to demonstrate a significant reduction in DB after duodenal EMR 6 . Recently, cold EMR has emerged as a potentially safer alternative to hot EMR. Nevertheless, concerns remain regarding higher recurrence rates and potential for muscularis propria damage 9 18 19 . Whether octreotide also could confer a benefit in patients with smaller lesions (< 30 mm) remains uncertain. Given the relatively low baseline risk of DB in this subgroup, which ranges from 3.1% to 7.3% 4 17 , the number needed to treat to achieve a meaningful clinical benefit is likely high. In these cases, in-hospital observation required for IV octreotide administration may not be justified. In contrast, closure of smaller post-EMR defects is typically feasible using TTS clips and may represent a more practical and cost-effective prophylactic measure.

The retrospective design, single-center setting, and limited number of patients treated with octreotide represent important limitations of this study. Potential risk of residual confounding—for example due to improved intraprocedural bleeding management over time—or time-dependent influence on DB occurrence cannot be ruled out. Nevertheless, these findings may serve as a foundation for future prospective studies to further evaluate the role of octreotide as a prophylactic agent. Such studies should also address comparisons with other prophylactic strategies and investigate optimal dosing, duration, and route of administration. Given that most DB cases occurred after octreotide discontinuation, it may be hypothesized that extending therapy beyond the initial 24-hour observation could further reduce risk of DB.

## Conclusions

**I** n conclusion, the results of our exploratory study suggest that 24-hour post-procedural IV octreotide was associated with a lower occurrence of DB after EMR of giant SNADTs (≥ 30 mm). Therefore, it may be a promising prophylactic option for this particular subgroup of patients at high risk of DB, for which well-established preventive strategies still remain limited.
